# Arboreal signals in the skeletal proportions of the *Gorilla* foot

**DOI:** 10.1111/joa.70259

**Published:** 2026-10-24

**Authors:** Elliot G. Greiner, Christopher B. Ruff, Andrea Lukova, Aventino Nkwasibwe, Thadee Muhire, Emmanuel Tibenda, Jason S. Massey, Juho‐Antti Junno, Samar M. Syeda, Jean Bosco Noheri, Fred Nizeyimana, Tierra Smiley Evans, Richard Muvunyi, Dennis Babaasa, Martha M. Robbins, Shannon C. McFarlin, Tracy L. Kivell

**Affiliations:** ^1^ Department of Human Origins Max Planck Institute for Evolutionary Anthropology Leipzig Germany; ^2^ Department of Anthropology Appalachian State University Boone North Carolina USA; ^3^ Center for Functional Anatomy and Evolution Johns Hopkins University School of Medicine Baltimore Maryland USA; ^4^ Department of Anthropology University of West Bohemia Pilsen Czech Republic; ^5^ Institute of Tropical Forest Conservation Mbarara University of Science and Technology Mbarara Uganda; ^6^ Dian Fossey Gorilla Fund Kinigi Rwanda; ^7^ Faculty of Medicine, Nursing, and Health Sciences Monash University Melbourne Australia; ^8^ Archeology University of Oulu Oulu Finland; ^9^ Department of Organismal Biology and Anatomy University of Chicago Chicago Illinois USA; ^10^ Division of Anthropology American Museum of Natural History New York New York USA; ^11^ Gorilla Doctors (Mountain Gorilla Veterinary Project, Inc.) Musanze Rwanda; ^12^ Gorilla Doctors (Mountain Gorilla Veterinary Project, Inc.) Kampala Uganda; ^13^ Gorilla Doctors (Mountain Gorilla Veterinary Project, Inc.) Davis California USA; ^14^ Department of Integrative Biology University of California Berkeley California USA; ^15^ Rwanda Development Board Kigali Rwanda; ^16^ Department of Primate Behavior and Evolution Max Planck Institute for Evolutionary Anthropology Leipzig Germany; ^17^ Department of Anthropology The George Washington University Washington DC USA

**Keywords:** arboreality, Bwindi, foot, gorilla, human, origins, Uganda

## Abstract

Postcranial skeletal proportions in living apes have been linked to patterns of arboreal behaviors within and between populations, offering a comparative baseline for interpreting arboreal behavior in fossil hominoids. We quantified metatarsal and proximal phalangeal lengths across all five pedal rays in *Gorilla*, comparing western lowland gorillas (*Gorilla gorilla gorilla*), low‐ and high‐elevation Grauer's gorillas (*Gorilla beringei graueri*), and two ecologically distinct mountain gorilla (*Gorilla beringei beringei*) populations: those of Bwindi Impenetrable National Park, Uganda, and the Virunga Massif (Volcanoes National Park, Rwanda). The Bwindi sample, assembled from individuals recovered by the Mountain Gorilla Skeletal Project between 2017 and 2025, represents the first substantial pedal dataset for this population. After separately scaling element lengths by estimated body mass and hindlimb length, Bwindi gorillas' third and fourth metatarsal lengths fell consistently between those of the more arboreal western lowland and highly terrestrial Virunga gorillas, while proximal phalangeal and hallux lengths varied little across populations. Additionally, Virunga gorillas showed longer second metatarsals compared to other populations, a feature we link to their strongly terrestrial locomotor habits. In comparing relative manual and pedal element lengths from the same individuals, metacarpals were relatively longer than metatarsals across all populations in rays 2–5, with this pattern reversed in the hallux; among these, only the second ray showed a significant interpopulation difference, with western lowland gorillas exhibiting relatively longer metacarpals than all other groups. Though interpopulation differences in the lengths of pedal elements were generally weaker than those found in the manual elements of the same individuals, these findings suggest that habitat‐associated variation in foot morphology can be detected between populations of gorilla subspecies, underscoring the value of fine‐grained, population‐level skeletal comparisons for refining locomotor inference in the hominoid and hominin fossil record.

## Graphical abstract

Differences in arboreal behavior among gorilla populations are correlated with the relative lengths of their pedal digital elements. Bwindi gorillas have third and fourth metatarsals relatively shorter than those of more arboreal western lowland gorillas and longer than those of more terrestrial Virunga gorillas, while hallux and proximal phalangeal lengths vary little. These findings provide a comparative basis for interpreting locomotion in fossil apes and hominins. Photo credit: Charlotte King.

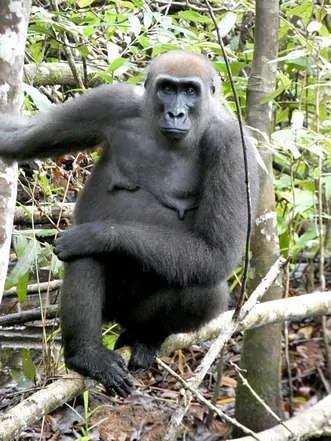

## 1 INTRODUCTION

In recent decades, research linking primate skeletal morphology to localized environmental contexts has deepened our understanding of the paleoecological relationships driving hominoid locomotor and behavioral evolution (Greiner et al., 2024; Hunt, 2016; Lukova et al., 2024, 2025; MacLatchy et al., 2023; Ruff et al., 2018, 2022). In particular, ecomorphometric differences in the hominoid skeleton have been identified within and between African ape populations (Dunn et al., 2014; Jabbour & Pearman, 2016; Knigge et al., 2015; Prang & Tocheri, 2024; Ruff et al., 2018, 2022; Taylor, 2006; Taylor & Groves, 2003; Tocheri et al., 2011), and include both cranial (Boughner & Dean, 2008; Humphrey et al., 1999; Taylor & Groves, 2003) and postcranial features (Knigge et al., 2015; Prang & Tocheri, 2024; Tocheri et al., 2011). Recent ecological and behavioral analyses of western lowland and mountain gorilla populations (Robbins et al., 2025) have also allowed for more fine‐grained ecomorphological investigations within mountain gorillas and between eastern and western gorillas, providing new insights into the selective pressures acting upon gorilla postcrania across populations.

Digital lengths in gorilla hands show marked inter‐ and intra‐(sub)specific differences associated with arboreality, as more arboreal populations tend to have longer metacarpals and proximal phalanges than more terrestrial populations, most pronounced in the central rays (2–4) (Greiner et al., 2026; Ruff et al., 2022). Though all gorillas are primarily terrestrial knuckle walkers, the longer manual digit elements seen in more arboreal gorillas are hypothesized to provide them with more surface area to support their body mass above ground, particularly when moving along tree branches (Greiner et al., 2026; King et al., 2026). While a similar pattern has been observed for the digit lengths of the gorilla foot, previous research is limited to the third ray (Ruff et al., 2022). Exploring the extent of this pattern across the pedal rays (i.e., rays 1–5) is important for clarifying (1) how arboreal and terrestrial substrates may influence pedal morphology in hominoids and (2) how integrated the hands and feet are in the context of ecomorphological adaptation. Together, this knowledge can help develop more robust ecomorphological datasets in which to interpret the hominoid and hominin fossil record.

Here, we build upon this previous work by investigating the ecomorphology of the gorilla foot (metatarsals and proximal phalanges of all five rays) and including the first extensive dataset of mountain gorillas (*Gorilla beringei beringei*) from the Bwindi Impenetrable National Park, Uganda. We focus on the potential morphological variation between the Bwindi and the only other mountain gorilla population, those of the Virunga Massif (with a focus on Volcanoes National Park, Rwanda), Uganda, and Democratic Republic of Congo, in comparison to western lowland and Grauer's gorillas, all of which live within notably different ecological settings. Our goal is to expand upon prior work (Greiner et al., 2026) that demonstrated an association between elongated fingers in *Gorilla* populations engaging in higher proportions of arboreal behaviors. By documenting relative lengths of pedal elements across multiple populations, we test whether this same pattern of digital lengthening/shortening is also present in the foot and subsequently evaluate the extent to which these metrics can distinguish ecologically driven behaviors at both inter‐ and intra‐(sub)specific levels. Ultimately, this approach aims to increase the analytical resolution available for reconstructing hominoid paleoecology.

### 1.1 Taxonomy and ecology of *Gorilla*

*Gorilla* comprises two species—*G. beringei* (eastern) and *G. gorilla* (western)—each with two subspecies: *G. b. beringei* (mountain) and *G. b. graueri* (Grauer's); *G. g. gorilla* (western lowland) and *G. g. diehli* (Cross River) (Xue et al., 2015). Eastern and western lineages diverged ~1.2–3 Ma, with intermittent gene flow occurring between some populations until ~14–10 ka (Städele et al., 2022; Thalmann et al., 2007; van der Valk et al., 2024). Within *G. beringei*, mountain and Grauer's gorillas diverged during the late Pleistocene (~14–12 ka), with prior population splits between Bwindi and Virunga groups (~26–20 ka) and later secondary gene flow (~5 ka), producing a paraphyletic relationship among mountain gorillas (Roy et al., 2014; Tocheri et al., 2016; van der Valk et al., 2024).

Western gorillas (excluding *G. g. diehli*, not considered here) inhabit dense, low‐elevation forests (<900 m) with seasonally variable fruit availability (Doran‐Sheehy et al., 2009; Gatti et al., 2004; Masi et al., 2009; Remis, 1997; Robbins et al., 2022). In contrast, eastern gorillas range from lowland tropical forests to the high‐elevation zones of Bwindi (1160–2600 m) and the Virunga Massif (2300–4500 m), with Grauer's gorillas occupying both low (<1300 m) and high (2100–2600 m) altitudes (Table 1; Mehlman, 2008).

**TABLE 1:** Observed rates of arboreality (as % of total observation time) and elevation ranges for analyzed gorilla populations. Taken from Greiner et al. (2026).

Population	Observed frequency of adult male (M) and female (F) arboreality	Habitat elevation
Western lowland gorilla (*G. g. gorilla*)	M = 19%, F = 34% (Loango, Gabon; Robbins et al., 2025)	<900 m
Low‐elevation Grauer's gorilla (*G*. *b. graueri*)	Not quantified	<1300 m
High‐elevation Grauer's gorilla (*G*. *b. graueri*)	Not quantified	~2100–2600 m
Bwindi mountain gorilla (*G. b. beringei*)	M = 18%, F = 21% (Robbins et al., 2025)	~1160–2600 m
Virunga mountain gorilla (*G. b. beringei*)	M = 2%, F = 7% (Doran, 1997)	~2300–4500 m

In Bwindi, mountain gorillas spend substantially more observed time in trees (females 21%, males 18%) than their Virunga counterparts (7% and 2%, respectively) (Doran, 1997; Robbins et al., 2025). Western lowland gorillas in Loango, Gabon, are relatively more arboreal (females 34%, males 19%) and have a more frugivorous diet (approximately 30% of feeding time) than mountain gorillas (Robbins et al., 2022, 2025). Although quantitative data are lacking for Grauer's gorillas, both low‐ and high‐elevation populations appear to be intermediate in their frequency of arboreal behaviors between the western lowland and Virunga mountain gorilla subspecies (Remis, 1995, 1999; Robbins et al., 2025; Ruff et al., 2018).

#### 1.1.1 The gorilla foot

The feet of extant great apes have a suite of morphological specializations for grasping that enable effective locomotion and foraging in structurally complex arboreal environments (e.g., Harper, 2020; Zeininger et al., 2022). All nonhuman great apes possess a divergent, opposable hallux and curved, relatively long pedal phalanges (compared to humans), which, combined with mobile midfoot joints, enhance prehensility and enable secure and effective grasping of arboreal branches and irregular substrates (DeSilva, 2010; Holowka & Lieberman, 2018; McHenry & Jones, 2006; Nyakatura, 2019). However, relative to *Pan* and *Pongo*, *Gorilla* shows slightly reduced curvature in the lateral toes (rays 2–5) and more robust but less mobile hallucal phalanges, consistent with a greater frequency of terrestrial locomotion (Congdon, 2012; Finestone et al., 2018; Holowka et al., 2017; Wareing, 2016).

Comparable interspecific distinctions extend to the hindfoot (tarsals), where recent comparative analyses suggest convergent evolution of features associated with arboreality in *Pan* and *Pongo*, whereas *Gorilla* exhibits morphology thought to be advantageous for terrestrial locomotion (McNutt et al., 2018; Prang, 2025). In particular, gorillas show hindfoot features suited to terrestrial support and propulsion, including a more concave plantar calcaneal surface and everted cuboid articular surface, contrasting with the comparatively deeper posterior pivot facet of the calcaneocuboid joint on the calcaneus in the more arboreal *Pongo* and *Pan* (Nozaki et al., 2021). Additionally, the elongated proximal tuberosity observed in *Gorilla* calcanei, in contrast to the shortened one in *Pan* and *Pongo*, is associated with differences in the frequency of terrestriality and arboreality seen among these three genera (McNutt et al., 2018).

However, while these patterns are broadly observed across great ape genera, the variable arboreal and terrestrial locomotor regimes among gorillas have also been linked to intrageneric differences in foot morphology that broadly reflect differences in midfoot mobility and stability across multiple pedal elements (Dunn et al., 2014; Harper, 2020; Harper et al., 2021; Ruff et al., 2022). In general, western gorillas exhibit morphologies associated with greater midfoot mobility and inversion capacity, whereas eastern gorillas, particularly the Virunga mountain gorillas, show features indicative of increased midfoot stability and axial loading. For instance, western gorilla calcanei exhibit relatively deeper posterior calcaneocuboid facets than the more terrestrial eastern gorillas. In comparison, the calcanei of Grauer's gorillas retain a deeper posterior calcaneocuboid facet, associated with greater midfoot mobility, while the more terrestrial Virunga mountain gorillas exhibit a flatter and shorter cuboid facet indicative of increased midfoot stability (Dunn et al., 2014; Harper, 2020; Harper et al., 2021; Jabbour & Pearman, 2016; Tocheri et al., 2011).

Metatarsal and phalangeal proportions also distinguish between eastern and western taxa in the foot, with western gorillas exhibiting relatively longer third metatarsals and proximal phalanges compared to eastern lowland gorillas, which would facilitate grasping of arboreal supports (Jabbour & Pearman, 2016; Ruff et al., 2022). Moreover, within eastern gorillas, Grauer's gorillas have a proportionally longer third pedal ray than the highly terrestrial Virunga mountain gorillas (Ruff et al., 2022). However, it remains unclear if these intrageneric and intraspecific differences within *Gorilla* are also found in the other pedal rays and how these potential proportional differences compare with those documented in *Gorilla* manual rays (Greiner et al., 2026). Furthermore, such proportions have not yet been evaluated among Bwindi mountain gorillas.

### 1.2 Predictions

We build upon previous studies linking hominid foot and hand morphology to locomotor behavior (e.g., Jabbour & Pearman, 2016; Ruff et al., 2018, 2022; Susman, 1979; reviewed in Kivell et al., 2023) and, more specifically, demonstrating correlations between digit length and arboreality in the *Gorilla* hand (Greiner et al., 2026). Here, we quantify the linear metrics of gorilla metatarsals and pedal proximal and intermediate phalanges across all five rays (Table 2) in western lowland, mountain (Bwindi and Virunga populations), and Grauer's (low‐ and high‐elevation populations) gorillas, to evaluate whether proportional variation among these taxa corresponds to differences in arboreal behavior (Table 1).

**TABLE 2:** Metatarsal and proximal phalangeal sample for each gorilla population and sex.

Bone	*G.b. beringei* (Bwindi)			*G.b. beringei* (Virunga)			*G.b. graueri* (low elevation)			*G.b. graueri* (high elevation)			*G.g. gorilla*		
	*N*	Male	Female	*N*	Male	Female	*N*	Male	Female	*N*	Male	Female	*N*	Male	Female
Metatarsal 1	22	13	9	36	17	19	0	0	0	11	7	4	13	8	5
Metatarsal 2	20	11	9	41	21	20	0	0	0	10	5	5	13	8	5
Metatarsal 3	20	11	9	44	23	21	2	1	1	12	7	5	57	29	28
Metatarsal 4	20	11	9	40	21	19	2	1	1	11	6	5	14	8	6
Metatarsal 5	20	11	9	38	20	18	2	1	1	11	6	5	14	8	6
Prox. Phalanx 1	18	10	8	28	13	15	0	0	0	7	4	3	13	8	5
Prox. Phalanx 2	13	8	5	26	13	13	0	0	0	4	2	2	12	7	5
Prox. Phalanx 3	13	8	5	30	16	14	0	0	0	4	2	2	13	8	5
Prox. Phalanx 4	12	5	7	27	14	13	1	1	0	3	1	2	12	8	4
Prox. Phalanx 5	10	6	4	31	17	14	1	0	1	6	2	4	12	8	4

We predict that if foot proportions are influenced by selection favoring arboreal locomotion, western lowland gorillas should display comparatively longer pedal rays relative to body mass or lower limb length than eastern gorilla species. This would reflect the functional integration of ape autopods, as well as the mechanical benefits of elongated digits for stability during above‐ground positional behaviors (Arlegi et al., 2025; King et al., 2026). Additionally, we predict that scaled inter‐ and intrasubspecific patterns in relative digital lengths will follow the patterns seen in the hands of these populations (Greiner et al., 2026), with western lowland gorillas having the longest lengths in rays 2–5 and Virunga mountain gorillas the shortest relative ray lengths, while Bwindi mountain gorillas will be more similar to western lowland gorillas and low‐ and high‐elevation Grauer's gorillas will exhibit greater overlap with Virunga gorillas.

Due to the abducted position of the hallux and its potential functional role in grasping behaviors in arboreal and uneven terrestrial contexts (such as rocky or densely vegetated terrain), we predict that the relative length of the first pedal ray will be more similar across populations than rays 2–5. Greiner et al. (2026) similarly found no clear length signal in the first manual ray of gorillas from these populations, which they attributed to the first digit's importance in manual grasping and manipulation, as well as the fact that the first manual ray is not loaded during knuckle‐walking. Although these functional explanations are specific to the hand, they could also suggest that the first ray may be subject to different selective or developmental constraints than the lateral rays in both the hand and foot.

Following Ruff et al. (2018, 2022) and Greiner et al. (2026), we hypothesize that gorilla groups will exhibit similar allometric slopes in foot bone scaling relative to body mass, but differ according to altitudinal range that is linked to frugivory, with more arboreal groups (western lowland and Bwindi mountain gorillas) showing relatively longer metatarsals and phalanges for a given body mass than more terrestrial groups (Virunga mountain and high‐elevation Grauer's gorillas). We further predict that, as in the hand, the first pedal ray may show a weaker relationship with arboreality than rays 2–5, potentially reflecting distinct selective or developmental constraints on the first ray. Among the lateral pedal rays, however, selection for elongation is expected to be reduced in more terrestrial populations because these rays are loaded during terrestrial locomotion (Tuttle, 1967).

Finally, because of the heightened importance of manual branch grasping relative to pedal branch grasping in arboreal gorillas (i.e., for stabilization in trees other than climbing), we predict that metacarpals will be relatively longer than the corresponding metatarsals in rays 2–5. We expect the pedal first ray to follow a different pattern, and potentially to be more similar in length to the manual first ray, given that both are important for secure grasping around arboreal supports.

## 2 METHODS

Excluding Bwindi mountain gorillas, adult *G. gorilla* and *G. beringei* third metatarsal (Mt3) and third proximal phalanx (PP3) measurements were obtained from a previous study by Ruff et al. (2022), which included data from the following curatorial institutions: The Field Museum of Natural History (Chicago, USA), Museum of Comparative Zoology (Cambridge, USA), Swedish Museum of Natural History (Stockholm, Sweden), The National Museum of Natural History (Washington, D.C.), The Powell Cotton Museum (Birchington, UK), The Royal Belgian Institute of Natural Science (Brussels, Belgium), The Royal Museum for Central Africa (Tervuren, Belgium), and the Mountain Gorilla Skeletal Project (MGSP) in Rwanda (Volcanoes National Park). The present study expanded this dataset by collecting length data for the other foot rays, using specimens from the National Museum of Natural History, the Royal Museum for Central Africa, and the MGSP. When possible, specimens collected for the present study were from the same individuals as in Ruff et al. (2022) in order to analyze the variation among digits. In addition, novel Bwindi mountain gorilla data for all five pedal rays were collected from the Institute for Tropical Forest Conservation (ITFC; Ruhija, Uganda), which serves as the repository for Bwindi mountain gorilla specimens, including specimens collected since 2017 by the MGSP.

Mountain gorilla skeletons curated by the MGSP were recovered postmortem (or catalogued, for pre‐existing specimens) from Volcanoes National Park in Rwanda (from 2007 to 2025) and Bwindi Impenetrable National Park in Uganda (from 2017 to 2025), in collaboration with Rwanda Development Board, Uganda Wildlife Authority, Gorilla Doctors, Dian Fossey Gorilla Fund, and other partners (McFarlin et al., 2009). Most of these skeletons were well‐preserved with relatively complete foot skeletons and often were from known individuals (e.g., with documented age and sex). Individuals were considered adult if the epiphyses of all elements were fully fused.

Digital caliper measurements were collected for maximum proximodistal length for all available metatarsals and proximal phalanges of the five pedal rays, following Ruff et al. (2022) (Table 2; Supporting Information: data sheet 1). All measurements were rounded to the tenth of a millimeter. Identification of the ray to which each phalanx belonged followed morphological descriptions in Zihlman and Underwood (2019). Because measurements were collected by multiple researchers (EG, TM, SS), including third metatarsal data previously published in Ruff et al. (2022), we conducted a calibration exercise to ensure consistency. Each researcher measured the third metatarsal length of at least ten specimens included in Ruff et al. (2022) and compared their results to the published values. Differences between observer and published values were calculated, and one‐sample tests were used to assess whether mean differences deviated significantly from zero, thereby evaluating the presence of systematic offsets. No significant differences were found, and therefore no adjustments were applied to any measurements.

Variation in relative ray lengths was evaluated among individual elements. All absolute lengths were first standardized by estimated body mass, with body mass calculated following the gorilla‐specific equations in Ruff et al. (2022) based on linear articular dimensions as predictor variables (described in Ruff et al., 2022; Ruff, 2007). In this study, the mediolateral articular breadth of the distal femur was used as the predictor variable as this was found to be the best estimator of body mass for gorillas with fully fused epiphyses (percent prediction error of 9.0%, Ruff et al., 2022; also see Burgess et al., 2018). Following our previous study of gorilla hand bones (Greiner et al., 2026), reduced major axis (RMA) regressions of log‐transformed lengths against body mass were run through the total pooled sample, and residuals from these regressions were used for comparisons between groups. Population comparisons were carried out using Kruskal‐Wallis and pairwise Wilcoxon tests (Holm‐adjusted) (Holm, 1979). Sample sizes differed for each skeletal element and across populations, and therefore we only ran analyses on samples with *n* ≥ 5 individuals. This eliminated all statistical analyses of low‐elevation Grauer's gorillas (Table 2), although they are still included in plots for visual comparison. We also calculated RMA regressions through each group and carried out comparisons of slopes and elevations between groups to further examine scaling patterns.

In addition to analyses using body mass as a size parameter, we evaluated metatarsal and phalangeal lengths relative to femoral+tibial length, to assess variation in foot relative to total limb length proportions, using similar statistical methods. These analyses were carried out within sex to account for known sex differences in limb proportions and to avoid conflating proportional variation in the foot with sexual dimorphism in hindlimb length, as well as to investigate potential sex‐specific differences in ray lengths independent of overall body size.

Pairwise contrasts of hand–foot effect sizes (Δlog length = metacarpal − metatarsal) were derived from estimated marginal means of log‐transformed metapodial lengths for homologous rays, incorporating metacarpal data derived from Greiner et al. (2026). Estimated marginal means were evaluated at the mean log(body mass) to control for body size differences. All pairwise comparisons among populations within each ray and among rays within each population were conducted with Tukey‐adjusted *p*‐values to control the family‐wise error rate across complete sets of pairwise contrasts.

All analyses were performed and visualized in R (v4.3.0) using *emmeans*, *smatr*, *ggplot2*, and *dplyr* packages. A probability value of 0.05 was considered statistically significant.

## 3 RESULTS

### 3.1 Metatarsal and proximal phalangeal length relative to body mass proxy

Figure 1 depicts results for residuals from RMA regressions of log‐transformed metatarsal length on log‐transformed body mass used to assess relative Mt1–Mt5 lengths across combined‐sex gorilla populations. Multiple significantly different relationships were found, with detailed results reported in Table 3. After Holm correction, Bwindi gorillas are significantly longer than western lowland gorillas and high‐elevation Grauer's gorillas, but significantly shorter than Virunga gorillas in Mt2. Western lowland gorillas are significantly longer than high‐elevation Grauer's and Virunga gorillas in Mt3 length. High‐elevation Grauer's gorillas are significantly shorter than Virunga gorillas in Mt1, Mt2, and Mt4. In Mt2, Virunga gorillas are significantly longer than both western lowland and high‐elevation Grauer's gorillas. Mt1 and Mt5 show broad overlap among populations, whereas Mt3 exhibits the clearest population differentiation.

**FIGURE 1:**
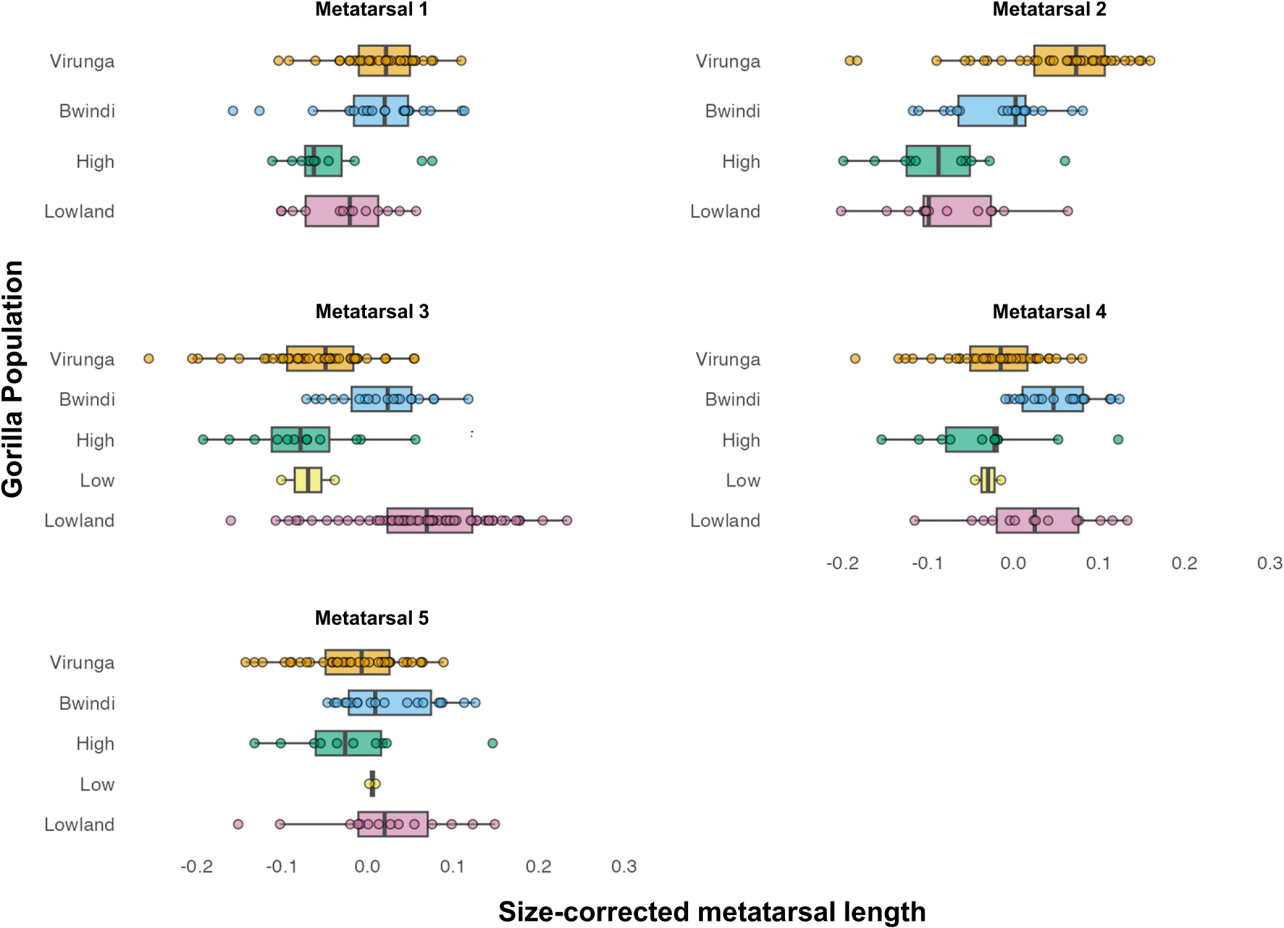
Metatarsal length relative to body mass (sexes pooled). Box and whisker plots of metatarsal length residuals derived from RMA regression of log(metatarsal length) on log(body mass). High refers to high‐elevation Grauer's gorillas, low to low‐elevation Grauer's gorillas, and lowland to western lowland gorillas.

**TABLE 3:** Summary of pairwise statistical comparisons among gorilla populations for metatarsal (Mt) and proximal phalanx (PP) lengths of each digital ray.

	Bwindi	Virunga	High G	Low G	Western low
Ray 1					
Bwindi					
Virunga					
High G		V*			
Western low					
Ray 2					
Bwindi		B**			B**
Virunga	V***(**)				
High G	B*(−)	V***			
Western low	B*(−)	V***(*)			
Ray 3					
Bwindi					
Virunga	B***(*)				
High G	B**(*)				
Western low	W*(−)	W***(**)	W***(*)		
Ray 4					
Bwindi		V**			W*
Virunga	B***(*)				
High G	B**(*)				
Western low		W(**)			
Ray 5					
Bwindi					
Virunga					
High G					
Western low		W(**)			

*Note*: For each ray, Mt. results are shown below the diagonal and PP results above the diagonal. Letter = group with longer element (residuals vs. body mass); Underline = also longer in line elevation tests; Parentheses = results differ when scaled to Femur + Tibia length, in level of significance, (−) = nonsignificant or reverses in direction, *p < 0.05; **p < 0.01; ***p < 0.001. Blank cells = nonsignificant comparisons, all analyses. ‘B’, Bwindi; ‘G’, Grauer, ‘V’, Virunga; ‘W’, western lowland gorillas.

Fewer significant differences were observed among populations in the pedal proximal phalanges than in the metatarsals, reflecting substantial overlap in size‐corrected residuals and reduced sample sizes for several elements (Figure 2). For PP1, no pairwise comparisons among populations are significant after Holm correction, indicating broadly overlapping proportional lengths across groups. In contrast, PP2 exhibits a significant difference between Bwindi and Virunga gorillas, with Bwindi gorillas showing relatively larger size‐corrected residuals. No significant differences were detected for PP3 in any pairwise comparison. For PP4, Bwindi gorillas are significantly shorter than Virunga gorillas, whereas length comparisons between Bwindi and high‐elevation Grauer's gorillas are not significant after correction. Finally, PP5 shows no significant differences among populations following Holm adjustment.

**FIGURE 2:**
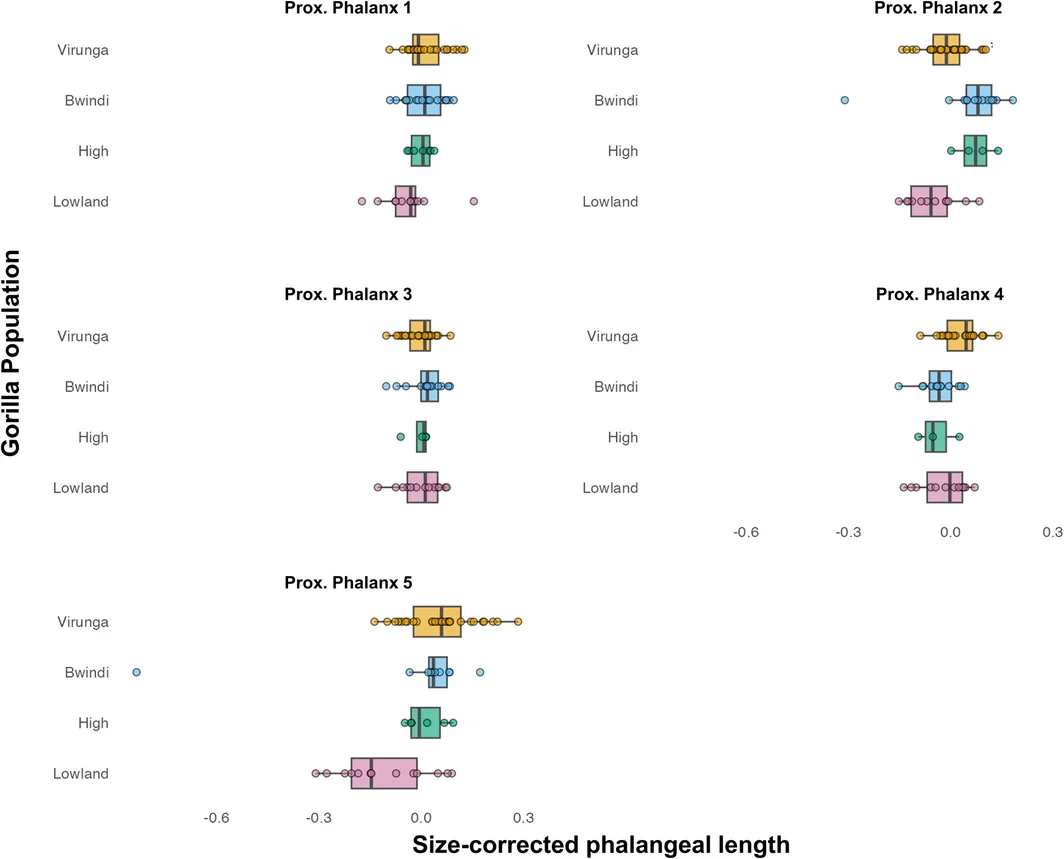
Phalangeal length relative to body mass (sexes pooled). Box and whisker plots of proximal phalangeal length residuals derived from RMA regression of log(phalangeal length) on log(body mass). High refers to high‐elevation Grauer's gorillas, low to low‐elevation Grauer's gorillas, and lowland to western lowland gorillas.

RMA regression slopes of log‐transformed metatarsal and phalangeal lengths against body mass within groups are generally negatively allometric, with slopes below the isometric expectation of 0.333 (Greiner et al., 2026; Ruff et al., 2022) (metatarsals: RMA slopes = 0.216–0.289; proximal phalanges: RMA slopes = 0.198–0.371). In metatarsals, differences in slopes across groups are nonsignificant (Table S1), indicating broad consistency in scaling patterns (see also Figure S1a). In contrast, line elevations differ significantly among groups for most metatarsals, with the strongest effects observed in Mts 2–4. Pairwise comparisons between groups demonstrated patterns broadly similar to those identified in RMA residual analyses (Tables S2 and S3). Proximal phalanges show a similar overall pattern of scaling consistency (Figure S1b), although significant slope heterogeneity was detected for PP2 and PP5. PP5 also exhibited the weakest mass–length correlation, suggesting greater variability in scaling behavior (Tables S1–S3). Differences in line elevations are significant for PP2, PP4, and PP5, but not for PP1 or PP3 (Table S1).

### 3.2 Metatarsal and proximal phalangeal length relative to lower limb length

To assess variation in foot bone proportions relative to lower limb size, we performed RMA regressions of log‐transformed metatarsal lengths (Mt1–Mt5) against log‐transformed combined tibial and femoral lengths, analyzed separately for males and females. Unlike estimated body mass, which captures overall body size but does not directly account for variation in limb segment proportions, combined tibial and femoral length provides a direct measure of hindlimb size, allowing metatarsal scaling relationships to be evaluated relative to the skeletal segments with which they functionally integrate. RMA regression was used to account for shared allometric scaling between pedal elements and hindlimb length, thereby isolating proportional differences independent of overall limb size. Residuals from these regressions were used for statistical comparisons among gorilla populations (Tables S4–S7; Figures 3, 4, 5, 6; Results for pooled sex comparisons are also shown in Table 3).

**FIGURE 3:**
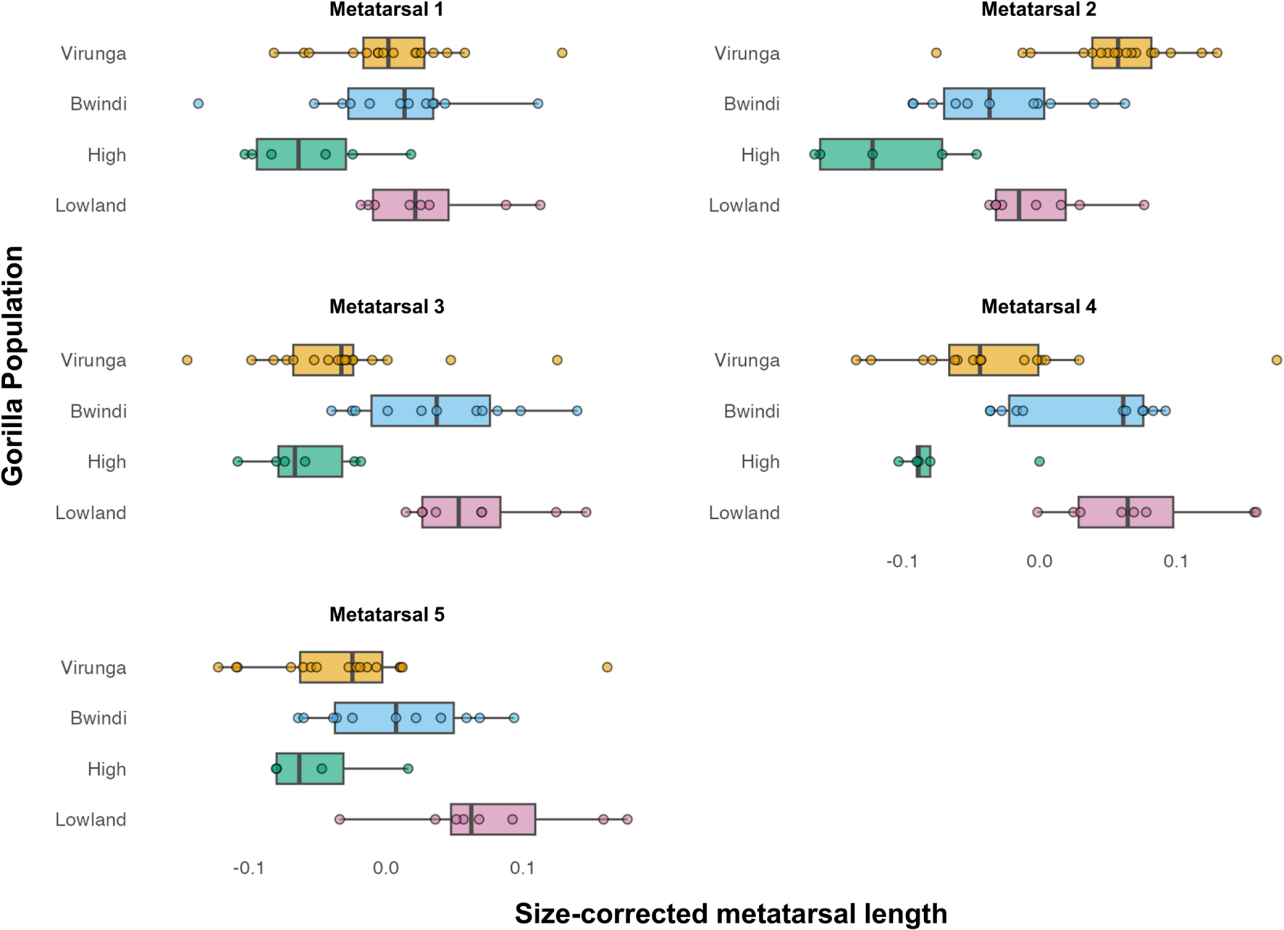
Metatarsal length relative to tibial + femoral length (male). Box and whisker plots of male metatarsal length residuals derived from RMA regression of log(metatarsal length) on log(tibial + femoral length). High refers to high‐elevation Grauer's gorillas, and lowland to western lowland gorillas.

Among males, western lowland and Bwindi gorillas possess significantly longer Mt3–Mt5 than Virunga males after Holm adjustment; differences between lowland and Bwindi males are small and nonsignificant across all rays (Figure 3; Table S4). Western lowland males are significantly longer than high‐elevation Grauer's males in Mt2–Mt4, and Bwindi males are also longer than high‐elevation Grauer's males in Mt3–Mt4. Virunga males have relatively longer Mt2's than all other groups. Among females, only western lowland and Bwindi gorillas have relatively longer Mt3 and Mt4 lengths than Virunga gorillas (Figure 4; Table S5). All remaining between‐population comparisons among females are not significant after Holm correction.

**FIGURE 4:**
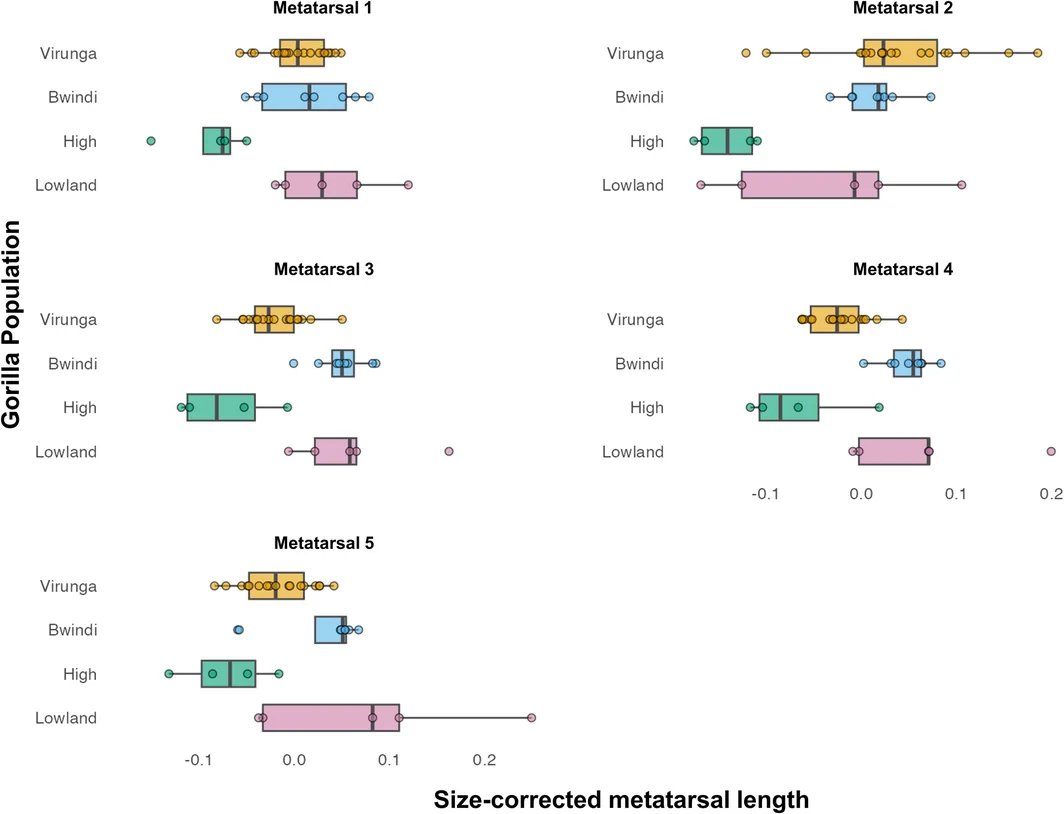
Metatarsal length relative to tibial + femoral length (female). Box and whisker plots of female metatarsal length residuals derived from RMA regression of log(metatarsal length) on log(tibial + femoral length). High refers to high‐elevation Grauer's gorillas, and lowland to western lowland gorillas.

Figures 5 and 6 depict residuals from RMA regressions of log‐transformed PP1–PP5 on log‐transformed tibial+femoral length, with results of pairwise comparisons given in Tables S6 and S7. Among males, relative proximal phalangeal proportions vary by ray, with no single population showing consistent elongation across all rays, typical of the comparisons reported above. Virunga males are not significantly longer than other populations in PP2, indicating that ray 2 elongation in this population is limited to the metatarsal rather than extending to the proximal phalanx. Western lowland males have significantly longer relative PP3 lengths than Virunga males, but not in other rays. Western lowland males are also significantly longer than Bwindi males in PP4. Among females, significant differences were detected in PP3 only, with western lowland females exhibiting significantly longer relative PP3 lengths than Virunga females; the difference between Bwindi and Virunga females approached but did not reach significance.

**FIGURE 5:**
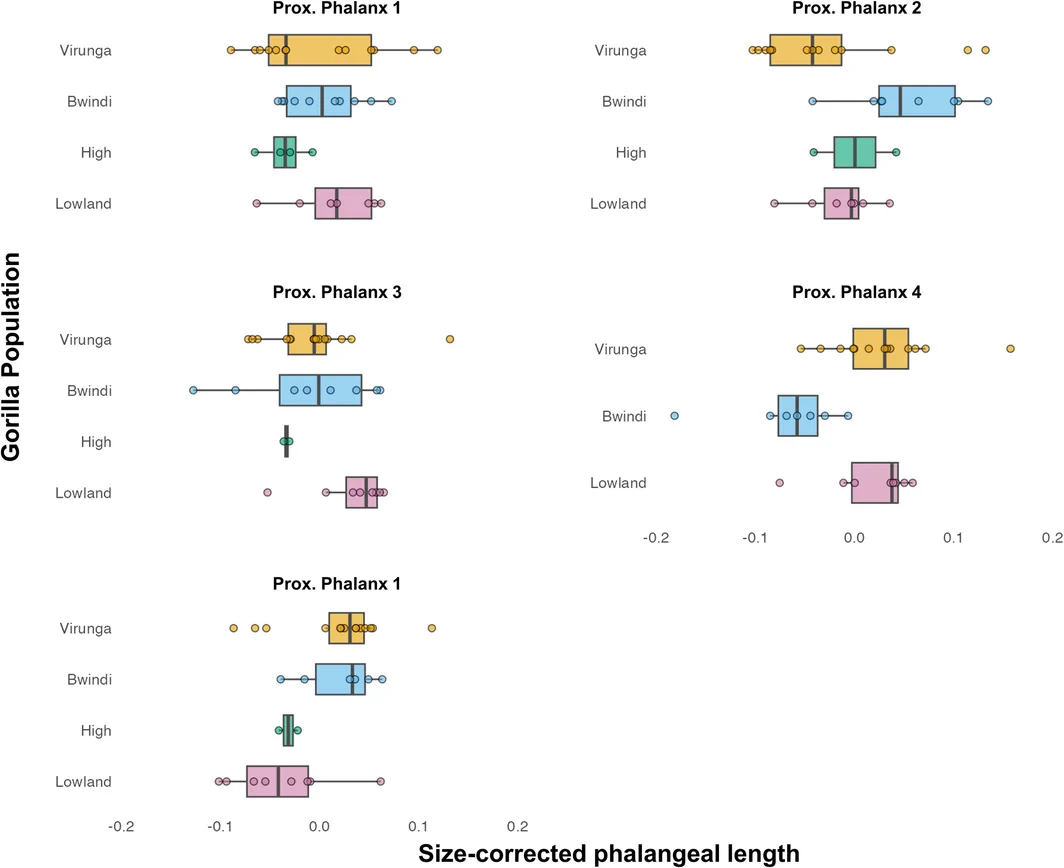
Proximal phalangeal length relative to tibial + femoral length (male). Box and whisker plots of male proximal phalangeal length residuals derived from RMA regression of log(phalangeal length) on log(tibial + femoral length). High refers to high‐elevation Grauer's gorillas, and lowland refers to western lowland gorillas.

**FIGURE 6:**
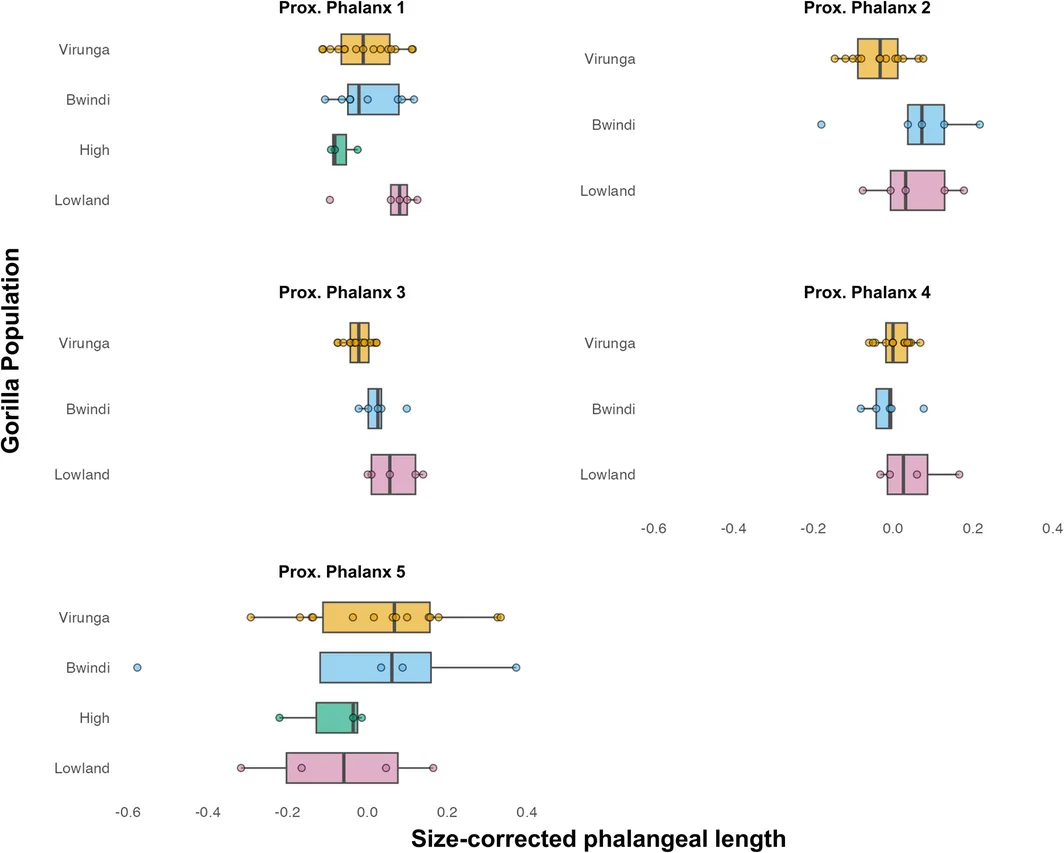
Proximal phalangeal length relative to tibial + femoral length (female). Box and whisker plots of female proximal phalangeal length residuals derived from RMA regression of log(phalangeal length) on log(tibial + femoral length). High refers to high‐elevation Grauer's gorillas, and lowland to western lowland gorillas.

### 3.3 Metacarpal and metatarsal comparison

Comparison of the relative length of the hand (Mc length) and foot (Mt length) ray elements, controlled for estimated body mass, shows that for metapodials 2–5, metacarpals are relatively longer than metatarsals across all populations (see Greiner et al., 2026 for hand data). This pattern is reversed across these populations in the first digit, where the metatarsal is relatively longer than the metacarpal (Figure 7).

**FIGURE 7:**
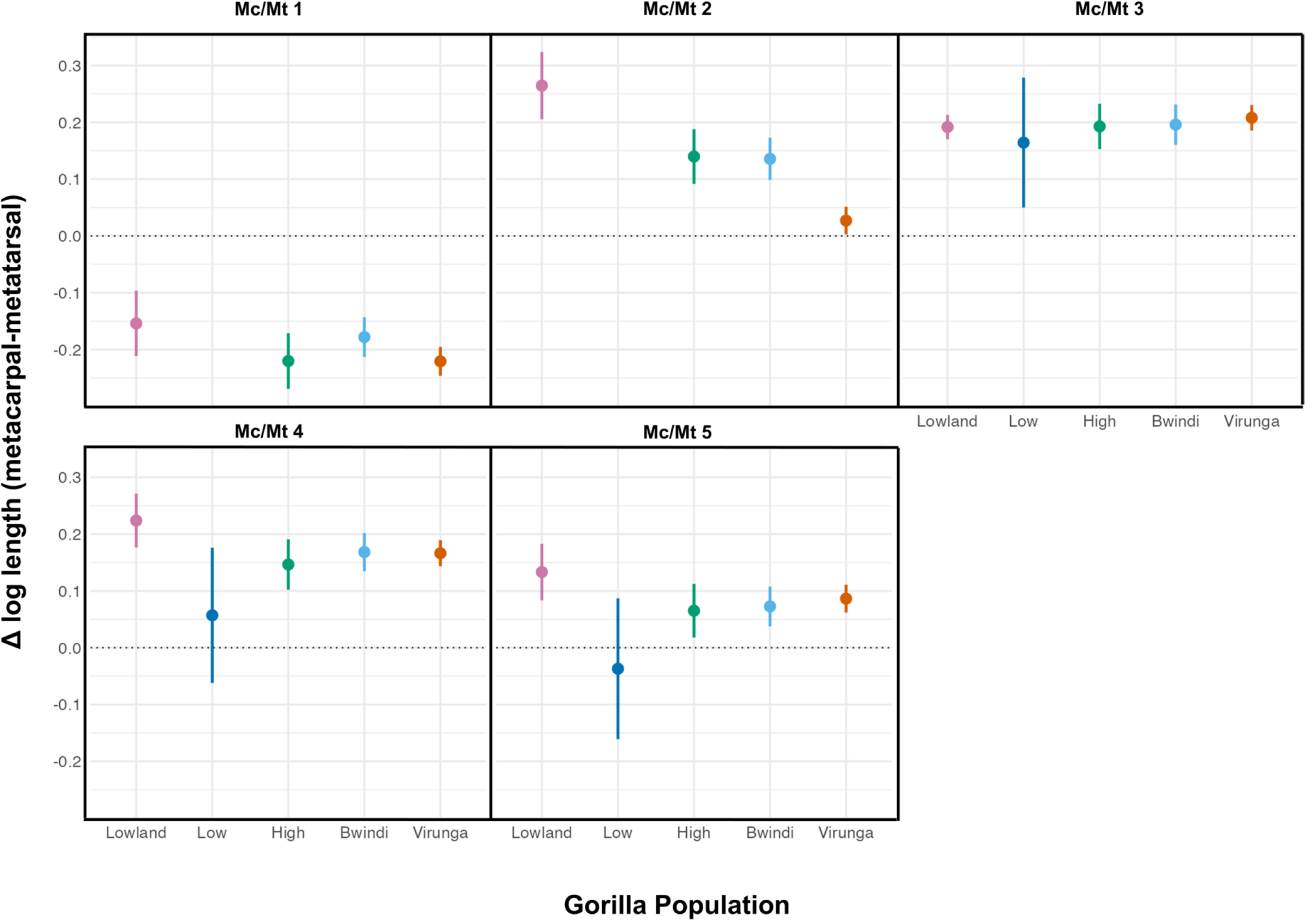
Metacarpal–Metatarsal effect sizes (Δ log length) across rays and populations. Estimated marginal means (±95% CI) for the difference in log‐transformed hand versus foot length (hand − foot), predicted at the mean log body mass. High refers to high‐elevation Grauer's gorillas, low to low‐elevation Grauer's gorillas, and lowland to western lowland gorillas.

Pairwise comparisons of hand–foot effect sizes (Δlog length = log[metacarpal] − log[metatarsal], evaluated at mean log body mass), in which positive values indicate relatively longer metacarpals than metatarsals, revealed interpopulation differences only for ray 2. Western lowland gorillas exhibited significantly greater positive Δlog values (i.e., relatively longer Mc2s) than all other groups (Table S8). In contract, Virunga gorillas has significantly lower Δlog values than high‐elevation Grauer's and Bwindi gorillas, indicating relatively longer Mt2 lengths.

In rays 3, 4, and 5, all pairwise comparisons were nonsignificant (all *p* ≥ 0.81).

## 4 DISCUSSION

Following Greiner et al. (2026), we predicted that western lowland and Bwindi gorillas, as the more arboreal populations, would have relatively longer metatarsals and proximal phalanges compared to the more terrestrial high‐elevation Grauer's and Virunga gorilla populations. Overall, our results for Mt3 and Mt4 support this prediction, with relatively longer elements in more arboreal western lowland and Bwindi gorillas than in Virunga and high‐elevation Grauer's gorillas, though Virunga gorillas exhibit relatively longer Mt2 than all other populations (discussed below). Bwindi gorillas are intermediate in relative length between Virunga and western lowland gorillas in the Mt3, the best sampled element. No similar trends are apparent in the corresponding proximal phalanges. We observed more similarities in hallux length between eastern and western populations, consistent with our prediction that the abducted position of this digit would obscure terrestrial/arboreal signals. Finally, supporting our prediction that manual elements would be relatively longer than pedal elements across populations, we found that in rays 2–5 manual digital elements were relatively longer than the pedal digital elements across all populations while this pattern was reversed in the first ray. These results are discussed in more detail below.

### 4.1 Differences in metatarsal and phalangeal relative lengths

The relatively longer 3rd and 4th (and, to a lesser extent, 5th) metatarsals exhibited by western lowland and Bwindi gorillas are consistent with their more frequent arboreal behaviors and the associated functional demands of foot‐to‐foot or foot‐to‐hand opposition during climbing. By contrast, the comparatively shorter Mt3–5 of Virunga and high‐elevation Grauer's gorillas, together with the relatively longer Mt2 of Virunga gorillas, likely reflect their documented (Doran, 1996, 1997; Robbins et al., 2025), as well as presumed, terrestrially dominated locomotor repertoires (see below). Similar to the pattern previously observed in the hands (Greiner et al., 2026), the metatarsal lengths of the Bwindi mountain gorillas are intermediate relative to the other populations for Mt3, the best sampled element. Notably, however, for Mt4–5, Bwindi gorilla lengths are very similar to those of western lowland gorillas when corrected for estimated body mass (see below for discussion of Mt1 results). The lengths of PP 1–5 show less consistent, and mostly nonsignificant, length differences between the gorilla populations when controlled for both estimated body mass and limb length. Notably, Grauer's gorillas were absent from some phalangeal comparisons and too poorly represented for statistical comparison with other groups (Figures 5, 6).

While the length differences between gorilla populations were not as pronounced in the foot as they were in the hand (Greiner et al., 2026), they show a similar correspondence with population and species‐level arboreality. We hypothesize that longer central pedal rays (Mt3–Mt4) would be advantageous for more arboreal gorillas because they would enhance the functional versatility of the foot during movement and support in an arboreal context (Cartmill, 1985). For gorillas and other arboreal primates, the difficulty of maintaining stability on an arboreal substrate increases with the degree of inclination, particularly on vertical supports (Cartmill, 1974; Preuschoft, 2002). During ascent, gorillas must generate sufficient limb forces to both overcome gravity and counter opposing shear forces, which elevate the risk of falling; this is especially risky if secure contact with the substrate cannot be maintained or facilitated (Cartmill, 1974; Granatosky et al., 2019; Hirasaki et al., 1993; Neufuss et al., 2018).

To compensate, gorillas typically use their forelimbs to pull and their hind limbs to push, and during descent increased braking forces in the foot are required to control acceleration and maintain stability (King et al., 2026; Neufuss et al., 2018; Preuschoft, 2002). In these efforts, elongated pedal rays increase the grasping capacity of the foot, providing greater stability and control when navigating or feeding in trees (Goodenberger et al., 2015; Moriyama, 1981). Additionally, longer central pedal rays also facilitate better weight distribution and balance, allowing the foot to adjust finely to narrow or flexible substrates during climbing, while reducing muscular effort (Neufuss et al., 2018; Susman & Stern, 1979).

However, when arboreal, the feet of gorillas are much more inert relative to their hands, which are often engaged in foraging behaviors or being relied on for support (King et al., 2026). Subsequently, the patterns seen in the relative lengths of the pedal digits, particularly the 1st, 2nd, and 5th, are weaker when compared to those seen in the manual digits (Figures 1 and 2). Relative to the hand rays, we infer that the foot's lessened function in arboreal grasping, coupled with its greater functional role in the generation of propulsive forces and weight support during terrestrial locomotion (McClymont et al., 2022; Prang, 2025; Simpson et al., 2018), results in decreased selective forces on pedal digital length. Consequently, length patterns in the foot might also relate to integration between pedal and digital elements, discussed more below.

We also hypothesize that the relatively longer Mt2 lengths observed in Virunga gorillas reflect selection associated with the population's highly terrestrial locomotor behavior. Because the hallux remains divergent and is not the primary propulsive digit, terrestrial walking likely shifts load‐bearing and propulsion to the second and third rays. This is consistent with comparative studies of ape forefoot biomechanics, in which force transmission is biased medially through the talonavicular–cuneiform complex and second metatarsal, such that a longer Mt2 may increase the lever arm during push‐off (Fernández et al., 2016), thereby improving propulsive efficiency and stability during toe‐off (Patel et al., 2018). Nevertheless, additional in vivo biomechanical and kinematic analyses are needed to evaluate how variation in metatarsal proportions influences load transmission and propulsion within and between gorilla populations.

#### 4.1.1 Relative length of the hallux

Considerable overlap is seen in the lengths of Mt1 and PP1 for all populations, supporting our prediction that the first ray would show less pronounced length differences between the gorilla populations due to hallucal mobility and its distinct functional roles relative to the other rays. When the hallux opposes the lateral digits around a narrow support, adduction may strengthen the grasp, as indicated by increased recruitment of the adductor hallucis during narrow‐support locomotion in other primates (Patel et al., 2015). On larger or vertical supports, hallucal position is more variable, and the hallux may remain abducted during climbing and descent (Prang, 2025; Sarmiento, 1994). The second and third toes, conversely, perform most of the fine, controlled flexion due to their stronger and more complex flexor muscles, which are optimized for generating force when the digits are already flexed, while the fourth and fifth toes provide flexion during grasping of vertical supports, aiding in maintaining grip and stability while climbing (Goh et al., 2019). Therefore, the functional variability of the first ray may obscure any clear relationship between length and the use of arboreal versus terrestrial substrates in the gorilla foot.

### 4.2 Compared manual and pedal ray lengths

We found that metatarsals and metacarpals share similar relative length patterns across gorilla populations, albeit the metatarsal patterns were less pronounced and, thus, showed fewer significant interpopulation differences than the metacarpals (Greiner et al., 2026). This finding is consistent with the idea that pedal digit lengths are under weaker directional selection for arboreality than the corresponding manual rays, despite shared underlying scaling relationships and developmental patterns (Rolian, 2016). Additionally, while the relatively elongate third and fourth metatarsals and phalanges of western lowland and Bwindi gorillas may represent a functional accommodation to efficient locomotion in both terrestrial and arboreal contexts (Robbins et al., 2025), the observed patterns of digital scaling in both hands and feet could indicate a degree of integration between homologous elements (Rolian, 2024). Such integration between corresponding manual and pedal rays would likely constrain evolutionary divergence between the hand and foot while still permitting differential ecological ‘tuning’, such as greater elongation of certain elements within the hand for climbing, and relative conservation in the foot for support and propulsion (Rolian et al., 2010; Young & Hallgrímsson, 2005). This limb‐wide modularity may explain why similar directional changes are observed in Mt3–Mt4 and their manual counterparts despite differing functional roles.

In the first ray, the metatarsal remains proportionally longer than the corresponding metacarpal across all populations. This offset primarily reflects an intrinsic morphological bias: in great apes, the hallux is consistently longer and more robust than the pollex (Shrewsbury et al., 2003; Szalay & Dagosto, 1988). Subsequently, the relative stability of this relationship across our gorilla populations suggests that increased arboreality does not substantially modify this pattern.

Shared developmental origins may also influence how much the pedal and manual digits can diverge. Early autopod development in mammals is orchestrated by conserved patterning systems, including Sonic Hedgehog (SHH)–FGF signaling and late‐phase HoxA/HoxD activity, which regulate digit number, identity, and distal growth in both fore‐ and hindlimb autopods (Pérez Gómez et al., 2018; Yakushiji‐Kaminatsui et al., 2018; Zakany & Duboule, 2007). Experimental manipulations of these pathways in mice can alter phenotypic expression in fore‐ and hindlimb digits; for instance, disruptions of Shh or posterior Hox genes generate parallel changes in digit number and relative size in both autopods (Chiang et al., 2001; Delpretti et al., 2012; Villavicencio‐Lorini et al., 2010). Furthermore, in chimpanzees, manual and pedal elements follow broadly similar, but not identical, developmental trajectories. Although metapodials and phalanges in both autopods scale positively with body mass during growth, manual elements generally grow for longer than their pedal counterparts, which may contribute to the stronger positive allometry of the hand (Combemorel et al., 2026). In gorillas, where the hands experience greater functional demands for grasping and climbing than the feet, a similar developmental scaffold could generate correlated elongation in corresponding digital rays while still permitting differences associated with the constraints of terrestrial locomotion. Thus, the covariation seen among homologous manual and pedal digits in western lowland and Bwindi gorillas may partly reflect conserved developmental mechanisms that constrain, but do not prevent, divergence between the hand and foot.

Additionally, and though not mutually exclusive, other factors, such as responses to climatic variation (consistent with Allen's Rule) might also contribute to interpopulation differences in digital proportions of the hands and feet (Strasser, 1992). Ruff et al. (2022) found that the distal limb segments of gorillas are shorter in higher elevation populations inhabiting colder temperatures, while the relative stability of distal proportions—particularly the phalanges—may subsequently reflect functional canalization of grasping digits against climatic effects. Though the authors also argue that the mechanical demands of terrestrial versus arboreal movement likely impose stronger selective pressures on the hands and feet than do temperature‐driven size reductions alone, the consistent phalangeal proportions observed across populations could indicate stabilizing selection maintaining grasping efficiency despite climatic variation, whereas differences in metacarpal dimensions may capture a more flexible balance between ecological conditions and locomotor adaptation.

## 5 CONCLUSIONS

Western lowland gorillas display longer relative metatarsal and phalangeal lengths across rays 3–4 than the more terrestrial Grauer's and Virunga gorillas, with the metatarsal lengths of the Bwindi mountain gorillas falling in between western and other eastern gorilla populations. However, these patterns are weaker relative to the those seen in the manual digital elements (Greiner et al., 2026). Taken together, we propose that the relatively longer metatarsals of the more arboreal western lowland and Bwindi mountain gorillas developed in concert with metacarpals to facilitate a larger contact point for propulsive force and braking during vertical climbing. The weaker patterns seen in the pedal elements might relate to a decreased reliance on foot stabilization in arboreal context, in addition to a functional trade‐off for efficient terrestrial walking and ‘toeing off’. Additionally, ray 2 lengths deviate from the patterns observed in the other digits in being generally longer in the Virunga mountain gorillas than in those from any other gorilla population. We interpret this as a consequence of their hyper‐terrestrial locomotor habits, as a longer Mt2 increases the mechanical advantage of the lever arm during push‐off.

In addition to locomotor behavior driving the length patterns noted here, it is also possible, and not mutually exclusive, that digital integration between the manual and pedal elements reflects broader developmental patterning shared across the autopod. Such integration could canalize covariation in relative segment lengths, even when the functional demands placed on the hands and feet differ. Under this view, selection acting on manual proportions to enhance arboreal performance, such as improved grasping efficiency or increased contact area during vertical climbing, may secondarily influence pedal proportions through shared genetic or developmental pathways. Conversely, the weaker expression of these patterns in the pedal rays may indicate that, although the hands and feet are developmentally linked, functional pressures on the hindlimb associated with terrestrial locomotion constrain the extent to which foot morphology can track manual adaptations.

Overall, these findings join others (Greiner et al., 2026; Jabbour & Pearman, 2016; Ruff et al., 2022) in highlighting the close relationship between gorilla skeletal morphology and localized ecology, emphasizing the importance of documenting population‐level environmental influences on hominoid anatomy and establishing rigorous extant ecomorphological baselines to better interpret variation observed in the fossil record (Greiner & Dagher, 2026).

## Supporting information


Data S1.

**Table S1.** SMA tests of slope heterogeneity and elevation (intercept) differences for log–log regressions of metatarsal and phalangeal maximum length against body mass across gorilla populations.
**Table S2.** Results of significant pairwise population (intercept) comparisons from standardized major axis (SMA) regressions of log metatarsal length on log body mass.
**Table S3.** Results of pairwise population (intercept) comparisons from standardized major axis (SMA) regressions of log proximal foot proximal phalanx length on log body mass.
**Table S4.** Pairwise comparisons of residual lengths for male metatarsals across gorilla populations (log [femoral+tibial length]).
**Table S5.** Pairwise comparisons of residual lengths for female metatarsals across gorilla populations (log [femoral+tibial length]).
**Table S6.** Pairwise comparisons of residual lengths for male proximal pedal phalanges across gorilla populations (log [femoral+tibial length]).
**Table S7.** Pairwise comparisons of residual lengths for female proximal pedal phalanges across gorilla populations (log [femoral+tibial length]).
**Table S8.** Pairwise population contrasts in Hand–Foot effect size (Δ log length) for each ray. For each ray, estimated marginal means of the Hand–Foot effect size (Δ log length), predicted at mean log body mass, were contrasted across all elevation groups. For every unordered population pair, the difference in Δ (Population₁ − Population_2_) was computed along with its standard error (SEdiffdiff). The table reports effect‐size differences (Estimate), their standard errors, and *p*‐values. Positive estimates indicate that Population 1 shows a larger Hand–Foot effect (greater relative hand elongation) than Population 2, whereas negative values indicate the reverse.
**Figure S1a.** Allometric scaling of gorilla metatarsals (Log[metatarsal length] vs. Log[Body Mass]).
**Figure S1b.** Allometric scaling of gorilla proximal pedal phalanges (Log[proximal phalangeal length] vs. Log[Body Mass]).

## Data Availability

The data that supports the findings of this study are available in the supplementary material of this article.
