# Supplementary material for: Arboreal signals in the skeletal proportions of the Gorilla foot
Source: J Anat. 2026 Oct 24:10.1111/joa.70259. Online ahead of print. doi: 10.1111/joa.70259 (PMC13695039; doi:10.1111/joa.70259)
Supplement: Supplementary file 1 [file JOA-9999-0-s001.docx]

**Supplement**

**Table S1**. SMA tests of slope heterogeneity and elevation (intercept) differences for log–log regressions of metatarsal and phalangeal maximum length against body mass across gorilla populations.

| **Bone** | **n** | **N groups** | **Slope p** | **Elev. p** |
| --- | --- | --- | --- | --- |
| MT1MAX | 82 | 4 | 0.320417 | 5.38E-02 |
| MT2MAX | 85 | 4 | 0.172385 | 1.25E-06 |
| MT3MAX | 135 | 5 | 0.440401 | 9.92E-19 |
| MT4MAX | 87 | 5 | 0.616141 | 1.44E-05 |
| MT5MAX | 85 | 5 | 0.450893 | 2.88E-02 |
| FPP1MAX | 66 | 4 | 0.187348 | 2.32E-01 |
| FPP2MAX | 56 | 4 | 0.038937 | 1.28E-02 |
| FPP3MAX | 60 | 4 | 0.348736 | 8.52E-01 |
| FPP4MAX | 54 | 4 | 0.596786 | 2.71E-03 |
| FPP5MAX | 58 | 5 | 0.000713 | 2.64E-03 |

**Table S2.** Results of significant pairwise population (intercept) comparisons from standardized major axis (SMA) regressions of log metatarsal length on log body mass.

| **Bone** | **pop_contrast** | **delta_resid** | **SE** | **df** | **t.ratio** | **p** |
| --- | --- | --- | --- | --- | --- | --- |
| MT1MAX | High - Virunga | -0.0543 | 0.01886 | 78 | -2.87892 | 2.59E-02 |
| MT2MAX | High - Virunga | -0.12867 | 0.026905 | 81 | -4.78229 | 4.48E-05 |
| MT2MAX | Lowland - Virunga | -0.11413 | 0.024255 | 81 | -4.70514 | 6.02E-05 |
| MT2MAX | Bwindi - Virunga | -0.05871 | 0.021111 | 81 | -2.78118 | 3.34E-02 |
| MT3MAX | Lowland - Virunga | 0.126371 | 0.0141 | 129 | 8.962484 | 2.59E-14 |
| MT3MAX | Lowland - High | 0.139907 | 0.022457 | 129 | 6.229886 | 3.65E-08 |
| MT3MAX | Bwindi - Virunga | 0.079769 | 0.019345 | 129 | 4.123519 | 3.83E-04 |
| MT3MAX | High - Bwindi | -0.09331 | 0.026072 | 129 | -3.57875 | 2.71E-03 |
| MT4MAX | Bwindi - Virunga | 0.073736 | 0.016092 | 81 | 4.582089 | 9.60E-05 |
| MT4MAX | High - Bwindi | -0.08265 | 0.021968 | 81 | -3.76207 | 1.77E-03 |
| MT4MAX | Lowland - Virunga | 0.052101 | 0.017949 | 81 | 2.902731 | 2.41E-02 |

**Table S3.** Results of pairwise population (intercept) comparisons from standardized major axis (SMA) regressions of log proximal foot proximal phalanx length on log body mass.

| **Bone** | **pop_contrast** | **delta_resid** | **p** |
| --- | --- | --- | --- |
| PP1 | Lowland - Virunga | -0.0501305 | 0.07961803 |
| PP1 | Lowland - Bwindi | -0.0458906 | 0.17424219 |
| PP1 | Lowland - High | -0.0375015 | 0.55012612 |
| PP1 | High - Virunga | -0.012629 | 0.95815287 |
| PP1 | Bwindi - High | 0.00838916 | 0.98889183 |
| PP1 | Bwindi - Virunga | -0.0042399 | 0.9953229 |
| PP2 | Lowland - Bwindi | -0.1082893 | 0.00921152 |
| PP2 | Lowland - High | -0.1235014 | 0.05563418 |
| PP2 | Bwindi - Virunga | 0.07017376 | 0.06607074 |
| PP2 | High - Virunga | 0.08538589 | 0.22267924 |
| PP2 | Lowland - Virunga | -0.0381156 | 0.54211234 |
| PP2 | Bwindi - High | -0.0152121 | 0.98805485 |
| PP3 | Bwindi - Virunga | 0.01491279 | 0.80588517 |
| PP3 | Lowland - Bwindi | -0.0135823 | 0.89957979 |
| PP3 | Bwindi - High | 0.01952787 | 0.90317982 |
| PP3 | Lowland - High | 0.0059456 | 0.99678383 |
| PP3 | High - Virunga | -0.0046151 | 0.99812602 |
| PP3 | Lowland - Virunga | 0.00133052 | 0.99981328 |
| PP4 | Bwindi - Virunga | -0.0654248 | 0.00942311 |
| PP4 | Lowland - Virunga | -0.0514392 | 0.05822388 |
| PP4 | High - Virunga | -0.0708559 | 0.18860912 |
| PP4 | Lowland - Bwindi | 0.01398561 | 0.93183619 |
| PP4 | Lowland - High | 0.0194167 | 0.95237251 |
| PP4 | Bwindi - High | 0.00543109 | 0.99884824 |
| PP5 | Lowland - Virunga | -0.1648581 | 0.01121245 |
| PP5 | Bwindi - Virunga | -0.0937715 | 0.34990133 |
| PP5 | Lowland - High | -0.1187321 | 0.40401886 |
| PP5 | Lowland - Bwindi | -0.0710866 | 0.68922027 |
| PP5 | High - Virunga | -0.046126 | 0.90748657 |
| PP5 | Bwindi - High | -0.0476455 | 0.93093459 |

**Table S4**. Pairwise comparisons of residual lengths for male metatarsals across gorilla populations (log [femoral+tibial length]).

| **Bone** | **Population 1** | **Population 2** | **n1** | **n2** | **Direction** | **p_adj** | **Significance** |
| --- | --- | --- | --- | --- | --- | --- | --- |
| Mt1 | Lowland | High | 8 |  |  |  |  |
| Mt1 | Lowland | Bwindi | 8 | 12 | > | 1 |  |
| Mt1 | Lowland | Virunga | 8 | 16 | > | 1 |  |
| Mt1 | High | Bwindi | 6 | 12 | < | 0.27119485 |  |
| Mt1 | High | Virunga | 6 | 16 | < | 0.08289088 |  |
| Mt1 | Bwindi | Virunga | 12 | 16 | > | 1 |  |
| Mt2 | Lowland | High | 8 | 5 | > | 0.01724143 | * |
| Mt2 | Lowland | Bwindi | 8 | 11 | > | 0.23119039 |  |
| Mt2 | Lowland | Virunga | 8 | 17 | < | 0.0398764 | * |
| Mt2 | High | Bwindi | 5 | 11 | < | 0.06271969 |  |
| Mt2 | High | Virunga | 5 | 17 | < | 0.0097392 | ** |
| Mt2 | Bwindi | Virunga | 11 | 17 | < | 0.0097392 | ** |
| Mt3 | Lowland | High | 8 | 6 | > | 0.01207325 | * |
| Mt3 | Lowland | Bwindi | 8 | 11 | > | 0.76295733 |  |
| Mt3 | Lowland | Virunga | 8 | 17 | > | 0.00598104 | ** |
| Mt3 | High | Bwindi | 6 | 11 | < | 0.01713693 | * |
| Mt3 | High | Virunga | 6 | 17 | < | 0.76295733 |  |
| Mt3 | Bwindi | Virunga | 11 | 17 | > | 0.01216475 | * |
| Mt4 | Lowland | High | 8 | 5 | > | 0.0338325 | * |
| Mt4 | Lowland | Bwindi | 8 | 11 | > | 0.26496794 |  |
| Mt4 | Lowland | Virunga | 8 | 16 | > | 0.00967229 | ** |
| Mt4 | High | Bwindi | 5 | 11 | < | 0.04417159 | * |
| Mt4 | High | Virunga | 5 | 16 | < | 0.25323011 |  |
| Mt4 | Bwindi | Virunga | 11 | 16 | > | 0.04417159 | * |
| Mt5 | Lowland | Bwindi | 8 | 11 | > | 0.10465038 |  |
| Mt5 | Lowland | Virunga | 8 | 16 | > | 0.00893381 | ** |
| Mt5 | Bwindi | Virunga | 11 | 16 | > | 0.145471 |  |

**Table S5.** Pairwise comparisons of residual lengths for female metatarsals across gorilla populations (log [femoral+tibial length]).

| **Bone** | **Population 1** | **Population 2** | **n1** | **n2** | **Direction** | **p_adj** | **Significance** |
| --- | --- | --- | --- | --- | --- | --- | --- |
| Mt1 | Lowland | Bwindi | 5 | 8 | > | 0.95899562 |  |
| Mt1 | Lowland | Virunga | 5 | 19 | > | 0.95899562 |  |
| Mt1 | Bwindi | Virunga | 8 | 19 | > | 0.95899562 |  |
| Mt2 | Lowland | Bwindi | 5 | 8 | < | 0.70552952 |  |
| Mt2 | Lowland | Virunga | 5 | 19 | < | 0.40653214 |  |
| Mt2 | Bwindi | Virunga | 8 | 19 | < | 0.70552952 |  |
| Mt3 | Lowland | Bwindi | 5 | 8 | > | 0.94165293 |  |
| Mt3 | Lowland | Virunga | 5 | 19 | > | 0.01113601 | * |
| Mt3 | Bwindi | Virunga | 8 | 19 | > | 0.0010143 | ** |
| Mt4 | Lowland | Bwindi | 5 | 8 | > | 0.8262002 |  |
| Mt4 | Lowland | Virunga | 5 | 18 | > | 0.02025308 | * |
| Mt4 | Bwindi | Virunga | 8 | 18 | > | 0.00073539 | *** |
| Mt5 | Lowland | Bwindi | 5 | 8 | > | 0.27319842 |  |
| Mt5 | Lowland | Virunga | 5 | 17 | > | 0.27319842 |  |
| Mt5 | Bwindi | Virunga | 8 | 17 | > | 0.086761 |  |

**Table S6.** Pairwise comparisons of residual lengths for male proximal pedal phalanges across gorilla populations (log [femoral+tibial length]).

| **Bone** | **Population 1** | **Population 2** | **n1** | **n2** | **Direction** | **p_adj** | **Significance** |
| --- | --- | --- | --- | --- | --- | --- | --- |
| PP1 | Lowland | Bwindi | 7 | 10 | > | 1 |  |
| PP1 | Lowland | Virunga | 7 | 13 | > | 1 |  |
| PP1 | Bwindi | Virunga | 10 | 13 | > | 1 |  |
| PP2 | Lowland | Bwindi | 7 | 8 | < | 0.07208301 |  |
| PP2 | Lowland | Virunga | 7 | 13 | > | 0.26725749 |  |
| PP2 | Bwindi | Virunga | 8 | 13 | > | 0.07208301 |  |
| PP3 | Lowland | Bwindi | 8 | 8 | > | 0.45429336 |  |
| PP3 | Lowland | Virunga | 8 | 15 | > | 0.04648289 | * |
| PP3 | Bwindi | Virunga | 8 | 15 | > | 0.72257201 |  |
| PP4 | Lowland | Bwindi | 8 | 7 | > | 0.02568253 | * |
| PP4 | Lowland | Virunga | 8 | 13 | > | 0.91349515 |  |
| PP4 | Bwindi | Virunga | 7 | 13 | < | 0.00599515 | ** |
| PP5 | Lowland | Bwindi | 8 | 6 | < | 0.16272226 |  |
| PP5 | Lowland | Virunga | 8 | 14 | < | 0.11211044 |  |
| PP5 | Bwindi | Virunga | 6 | 14 | > | 0.901539 |  |

**Table S7**. Pairwise comparisons of residual lengths for female proximal pedal phalanges across gorilla populations (log [femoral+tibial length]).

| **Bone** | **Population 1** | **Population 2** | **n1** | **n2** | **direction** | **p_adj** | **signif** |
| --- | --- | --- | --- | --- | --- | --- | --- |
| PP1 | Lowland | Bwindi | 5 | 8 | > | 0.68269863 |  |
| PP1 | Lowland | Virunga | 5 | 15 | > | 0.48760664 |  |
| PP1 | Bwindi | Virunga | 8 | 15 | < | 0.68269863 |  |
| PP2 | Lowland | Bwindi | 5 | 5 | < | 0.83453162 |  |
| PP2 | Lowland | Virunga | 5 | 13 | > | 0.28136869 |  |
| PP2 | Bwindi | Virunga | 5 | 13 | > | 0.28136869 |  |
| PP3 | Lowland | Bwindi | 5 | 5 | > | 0.4033953 |  |
| PP3 | Lowland | Virunga | 5 | 14 | > | 0.03268906 | * |
| PP3 | Bwindi | Virunga | 5 | 14 | > | 0.05915881 |  |
| PP4 | Bwindi | Virunga | 5 | 13 | < | 0.43035607 |  |

**Table S8.** **Pairwise population contrasts in Hand–Foot effect size (Δ log length) for each ray.**
For each ray, estimated marginal means of the Hand–Foot effect size (Δ log length), predicted at mean log body mass, were contrasted across all elevation groups. For every unordered population pair, the difference in Δ (Population₁ − Population₂) was computed along with its standard error (SEdiffdiff). The table reports effect-size differences (Estimate), their standard errors, and p-values. Positive estimates indicate that Population 1 shows a larger Hand–Foot effect (greater relative hand elongation) than Population 2, whereas negative values indicate the reverse.

| **Populations** | **Ray** | **delta_diff** | **SE** | **df** | **p** |
| --- | --- | --- | --- | --- | --- |
| (Hand - Foot Lowland) - (Hand - Foot Low) | Ray 1 | *NA* | *NA* | *NA* | *NA* |
| (Hand - Foot Lowland) - (Hand - Foot High) | Ray 1 | 0.06572205 | 0.03778382 | 924 | 3.04E-01 |
| (Hand - Foot Lowland) - (Hand - Foot Bwindi) | Ray 1 | 0.02394058 | 0.0335635 | 924 | 8.92E-01 |
| (Hand - Foot Lowland) - (Hand - Foot Virunga) | Ray 1 | 0.06651315 | 0.03157093 | 924 | 1.52E-01 |
| (Hand - Foot Low) - (Hand - Foot High) | Ray 1 | *NA* | *NA* | *NA* | *NA* |
| (Hand - Foot Low) - (Hand - Foot Bwindi) | Ray 1 | *NA* | *NA* | *NA* | *NA* |
| (Hand - Foot Low) - (Hand - Foot Virunga) | Ray 1 | *NA* | *NA* | *NA* | *NA* |
| (Hand - Foot High) - (Hand - Foot Bwindi) | Ray 1 | -0.0417815 | 0.02989982 | 924 | 5.01E-01 |
| (Hand - Foot High) - (Hand - Foot Virunga) | Ray 1 | 0.0007911 | 0.02764442 | 924 | 1.00E+00 |
| (Hand - Foot Bwindi) - (Hand - Foot Virunga) | Ray 1 | 0.04257258 | 0.02151989 | 924 | 1.97E-01 |
| (Hand - Foot Lowland) - (Hand - Foot Low) | Ray 2 | *NA* | *NA* | *NA* | *NA* |
| (Hand - Foot Lowland) - (Hand - Foot High) | Ray 2 | 0.12494516 | 0.03774934 | 924 | 5.35E-03 |
| (Hand - Foot Lowland) - (Hand - Foot Bwindi) | Ray 2 | 0.12944819 | 0.03459589 | 924 | 1.11E-03 |
| (Hand - Foot Lowland) - (Hand - Foot Virunga) | Ray 2 | 0.24310593 | 0.0317834 | 924 | 5.05E-13 |
| (Hand - Foot Low) - (Hand - Foot High) | Ray 2 | *NA* | *NA* | *NA* | *NA* |
| (Hand - Foot Low) - (Hand - Foot Bwindi) | Ray 2 | *NA* | *NA* | *NA* | *NA* |
| (Hand - Foot Low) - (Hand - Foot Virunga) | Ray 2 | *NA* | *NA* | *NA* | *NA* |
| (Hand - Foot High) - (Hand - Foot Bwindi) | Ray 2 | 0.00450303 | 0.02994004 | 924 | 9.99E-01 |
| (Hand - Foot High) - (Hand - Foot Virunga) | Ray 2 | 0.11816077 | 0.02664047 | 924 | 6.08E-05 |
| (Hand - Foot Bwindi) - (Hand - Foot Virunga) | Ray 2 | 0.11365773 | 0.02194488 | 924 | 1.63E-06 |
| (Hand - Foot Lowland) - (Hand - Foot Low) | Ray 3 | 0.02770901 | 0.05932536 | 924 | 9.90E-01 |
| (Hand - Foot Lowland) - (Hand - Foot High) | Ray 3 | -0.0016634 | 0.02288528 | 924 | 1.00E+00 |
| (Hand - Foot Lowland) - (Hand - Foot Bwindi) | Ray 3 | -0.0044253 | 0.020839 | 924 | 1.00E+00 |
| (Hand - Foot Lowland) - (Hand - Foot Virunga) | Ray 3 | -0.017316 | 0.01572008 | 924 | 8.06E-01 |
| (Hand - Foot Low) - (Hand - Foot High) | Ray 3 | -0.0293724 | 0.0617138 | 924 | 9.90E-01 |
| (Hand - Foot Low) - (Hand - Foot Bwindi) | Ray 3 | -0.0321344 | 0.0609846 | 924 | 9.85E-01 |
| (Hand - Foot Low) - (Hand - Foot Virunga) | Ray 3 | -0.045025 | 0.05943045 | 924 | 9.43E-01 |
| (Hand - Foot High) - (Hand - Foot Bwindi) | Ray 3 | -0.002762 | 0.02689535 | 924 | 1.00E+00 |
| (Hand - Foot High) - (Hand - Foot Virunga) | Ray 3 | -0.0156527 | 0.02315635 | 924 | 9.62E-01 |
| (Hand - Foot Bwindi) - (Hand - Foot Virunga) | Ray 3 | -0.0128907 | 0.02113633 | 924 | 9.74E-01 |
| (Hand - Foot Lowland) - (Hand - Foot Low) | Ray 4 | 0.16777593 | 0.06905838 | 924 | 1.08E-01 |
| (Hand - Foot Lowland) - (Hand - Foot High) | Ray 4 | 0.07728961 | 0.03469203 | 924 | 1.70E-01 |
| (Hand - Foot Lowland) - (Hand - Foot Bwindi) | Ray 4 | 0.05557063 | 0.03102528 | 924 | 3.79E-01 |
| (Hand - Foot Lowland) - (Hand - Foot Virunga) | Ray 4 | 0.05573906 | 0.02824941 | 924 | 2.80E-01 |
| (Hand - Foot Low) - (Hand - Foot High) | Ray 4 | -0.0904863 | 0.06842039 | 924 | 6.77E-01 |
| (Hand - Foot Low) - (Hand - Foot Bwindi) | Ray 4 | -0.1122053 | 0.06663617 | 924 | 4.44E-01 |
| (Hand - Foot Low) - (Hand - Foot Virunga) | Ray 4 | -0.1120369 | 0.06538992 | 924 | 4.26E-01 |
| (Hand - Foot High) - (Hand - Foot Bwindi) | Ray 4 | -0.021719 | 0.02957798 | 924 | 9.48E-01 |
| (Hand - Foot High) - (Hand - Foot Virunga) | Ray 4 | -0.0215506 | 0.02665179 | 924 | 9.28E-01 |
| (Hand - Foot Bwindi) - (Hand - Foot Virunga) | Ray 4 | 0.00016842 | 0.02166446 | 924 | 1.00E+00 |
| (Hand - Foot Lowland) - (Hand - Foot Low) | Ray 5 | 0.17035468 | 0.06912918 | 924 | 9.98E-02 |
| (Hand - Foot Lowland) - (Hand - Foot High) | Ray 5 | 0.06797551 | 0.03525701 | 924 | 3.03E-01 |
| (Hand - Foot Lowland) - (Hand - Foot Bwindi) | Ray 5 | 0.06085113 | 0.03118497 | 924 | 2.91E-01 |
| (Hand - Foot Lowland) - (Hand - Foot Virunga) | Ray 5 | 0.04554818 | 0.02855545 | 924 | 5.01E-01 |
| (Hand - Foot Low) - (Hand - Foot High) | Ray 5 | -0.1023792 | 0.06863735 | 924 | 5.68E-01 |
| (Hand - Foot Low) - (Hand - Foot Bwindi) | Ray 5 | -0.1095035 | 0.06663731 | 924 | 4.70E-01 |
| (Hand - Foot Low) - (Hand - Foot Virunga) | Ray 5 | -0.1248065 | 0.06544801 | 924 | 3.14E-01 |
| (Hand - Foot High) - (Hand - Foot Bwindi) | Ray 5 | -0.0071244 | 0.03007897 | 924 | 9.99E-01 |
| (Hand - Foot High) - (Hand - Foot Virunga) | Ray 5 | -0.0224273 | 0.02734329 | 924 | 9.24E-01 |
| (Hand - Foot Bwindi) - (Hand - Foot Virunga) | Ray 5 | -0.015303 | 0.02184264 | 924 | 9.56E-01 |

**Figure S1a.** **Allometric scaling of gorilla metatarsals (Log[metatarsal length] vs. Log[Body Mass])**

**Figure S1b. Allometric scaling of gorilla proximal pedal phalanges (Log[proximal phalangeal length] vs. Log[Body Mass])**
